# Value contribution of blood-based neurofilament light chain as a biomarker in multiple sclerosis using multi-criteria decision analysis

**DOI:** 10.3389/fpubh.2024.1397845

**Published:** 2024-04-22

**Authors:** Enric Monreal, Pilar Díaz Ruiz, Isabel López San Román, Alfredo Rodríguez-Antigüedad, Miguel Ángel Moya-Molina, Ana Álvarez, Elena García-Arcelay, Jorge Maurino, John Shepherd, Álvaro Pérez Cabrera, Luisa María Villar

**Affiliations:** ^1^Department of Neurology, Hospital Universitario Ramón y Cajal, Instituto Ramón y Cajal de Investigación Sanitaria, Red Española de Esclerosis Múltiple, Red de Enfermedades Inflamatorias, Universidad de Alcalá, Madrid, Spain; ^2^Department of Pharmacy, Hospital Nuestra Señora de Candelaria, Tenerife, Spain; ^3^Servicio de Salud de Castilla-La Mancha (SESCAM), Albacete, Spain; ^4^Department of Neurology, Hospital de Cruces, Bilbao, Spain; ^5^Hospital Universitario Puerta del Mar, Cádiz, Spain; ^6^Roche Farma, Madrid, Spain; ^7^Omakase Consulting, Barcelona, Spain; ^8^Department of Immunology, Hospital Universitario Ramón y Cajal, IRYCIS, Madrid, Spain

**Keywords:** biomarker, neurofilaments, inflammation, neurodegeneration, treatment response, multiple sclerosis (MS), multi-criteria decision analysis (MCDA)

## Abstract

**Introduction:**

Multiple sclerosis (MS) is a chronic autoimmune demyelinating disease that represents a leading cause of non-traumatic disability among young and middle-aged adults. MS is characterized by neurodegeneration caused by axonal injury. Current clinical and radiological markers often lack the sensitivity and specificity required to detect inflammatory activity and neurodegeneration, highlighting the need for better approaches. After neuronal injury, neurofilament light chains (NfL) are released into the cerebrospinal fluid, and eventually into blood. Thus, blood-based NfL could be used as a potential biomarker for inflammatory activity, neurodegeneration, and treatment response in MS. The objective of this study was to determine the value contribution of blood-based NfL as a biomarker in MS in Spain using the Multi-Criteria Decision Analysis (MCDA) methodology.

**Materials and methods:**

A literature review was performed, and the results were synthesized in the evidence matrix following the criteria included in the MCDA framework. The study was conducted by a multidisciplinary group of six experts. Participants were trained in MCDA and scored the evidence matrix. Results were analyzed and discussed in a group meeting through reflective MCDA discussion methodology.

**Results:**

MS was considered a severe condition as it is associated with significant disability. There are unmet needs in MS as a disease, but also in terms of biomarkers since no blood biomarker is available in clinical practice to determine disease activity, prognostic assessment, and response to treatment. The results of the present study suggest that quantification of blood-based NfL may represent a safe option to determine inflammation, neurodegeneration, and response to treatments in clinical practice, as well as to complement data to improve the sensitivity of the diagnosis. Participants considered that blood-based NfL could result in a lower use of expensive tests such as magnetic resonance imaging scans and could provide cost-savings by avoiding ineffective treatments. Lower indirect costs could also be expected due to a lower impact of disability consequences. Overall, blood-based NfL measurement is supported by high-quality evidence.

**Conclusion:**

Based on MCDA methodology and the experience of a multidisciplinary group of six stakeholders, blood-based NfL measurement might represent a high-value-option for the management of MS in Spain.

## 1 Introduction

Multiple sclerosis (MS) is a chronic autoimmune demyelinating disease of the central nervous system affecting over 2.8 million worldwide, often manifesting in adults aged 20–40, with women affected more than men. Its unpredictable symptoms, like fatigue, impair mobility and quality of life and cognitive dysfunction posing burdens on individuals, families, and healthcare systems (1, 2). It is the most common cause of non-traumatic disability in young and middle-aged adults (3, 4). MS is characterized by neurodegeneration caused by axonal injury, present from the early disease stages (5, 6). Due to the high variability of MS, in which the disease can manifest very differently between individuals and over time in the same patient, clinical and radiological markers may not be specific or sensitive enough to capture the full range of changes in terms of inflammation and neurodegeneration (7). Assessment and quantification of inflammatory activity and neurodegeneration are essential to establish the severity of the disease, the long-term prognosis, the need for treatment, the treatment option and the individual response to the selected treatment, as well as the achievement of therapeutic goals (7, 8).

The neuronal cytoskeleton is composed of actin, microtubules and neurofilaments (9). Neurofilaments are mainly located in myelinated axons, where they help maintain axonal structure and enable high-speed nerve conduction. Extracellular secretion of neurofilaments from the neuronal cytoskeleton has been registered in the context of axonal injury and neurodegeneration. Neurofilaments, once released into the extracellular space, reach the cerebrospinal fluid (CSF) and bloodstream. Light and heavy chains are sufficiently stable to be detected in blood by immunoassay and light chains allow longitudinal blood determination in clinical practice with high sensitivity and by a minimally invasive procedure (9). In this context, the assessment of blood-based neurofilament light chain (blood-based NfL) concentration in MS is becoming a practical tool for predicting clinical outcomes and monitoring subclinical disease activity in response to treatment (9–11). Indeed, elevated levels of blood-based NfL have been related to inflammatory activity, in terms of occurrence and severity of clinical relapses and increased frequency of lesions (12). Additionally, elevated levels of blood-based NfL have been associated with neurodegeneration, in terms of progression of physical and cognitive disability, as well as brain atrophy (5). Besides, assessment of the response to MS treatments could be another application of quantification of blood-based NfL and, in the future, could facilitate treatment choice in high-risk patients as a marker of response to treatment (5, 9, 13–17). Nevertheless, one of the significant challenges hindering the widespread utilization of NfL lies in its non-specific nature. Unlike some biomarkers that exhibit a high degree of specificity to specific pathological processes or diseases, NfL levels can be influenced by various factors beyond MS, including other neurodegenerative conditions, acute neurological insults, and even non-neurological disorders. This lack of specificity poses a critical hurdle in interpreting NfL measurements accurately and underscores the importance of contextualizing its levels within the broader clinical and pathological landscape (18–20).

Other blood and cerebrospinal fluid biomarkers are also being studied as biomarkers in MS (21). Particularly, there is growing interest in a novel blood biomarker known as glial fibrillary acidic protein (GFAP) in the field of neurological diseases (22). Its potential complementary role alongside blood-based NfL could significantly enhance prognostication and the development of disease management strategies for MS (23).

Multi-Criteria Decision Analysis (MCDA) enables determination of the value contribution of a health technology from the perspective of all stakeholders (clinicians, hospital pharmacists, patients, evaluators/payers, hospital directors and regional healthcare directors), stimulating structured discussions among all of them through an explicit set of quantitative and qualitative criteria (24, 25). This systematic, structured, objective, and transparent process allows for a more complete analysis of the overall value. It also provides arguments for decision-making, considering all criteria relevant in health care evaluation and decision-making, beyond the traditional assessments based on efficacy, safety, and cost (26).

The objective of this study was to determine the value contribution of blood-based NfL as a biomarker in MS in Spain using the MCDA methodology.

## 2 Materials and methods

### 2.1 Study design

The study was designed following good practice recommendations for MCDA methodology with the following structure (27, 28): literature review, evidence matrix development, criteria scoring, aggregate scoring, value determination, and discussion of findings.

The current study analyzed the value contribution of blood-based NfL as a biomarker in MS, using the Evidence and Value: Impact on Decision-Making (EVIDEM) MCDA framework previously adapted to MS (29). No specific framework for biomarkers in MS was found. The MS EVIDEM framework was then adapted to evaluate biomarkers in MS. An ideal biomarker in MS is considered to improve the sensitivity and specificity of diagnosis and to determine disease activity, neurodegeneration, and treatment response (5, 9). Therefore, the efficacy criterion was subdivided into these same sub-criteria: determination of MS diagnosis, evaluation of MS activity, assessment of neurodegeneration and detection of treatment response. Each sub-criterion was scored individually. The type of therapeutic benefit was not included since the present study aims to determine the value contribution of a biomarker (not a therapy) in MS. Acquisition costs and other direct costs were included in the same criterion.

The adapted framework used in the present study is shown in Table 1. The matrix includes: criteria related to MS (severity of the disease, population size and unmet needs), criteria related to blood-based NfL (efficacy/effectiveness, safety/tolerability, patient-reported outcomes, direct cost, non-medical/indirect costs, quality of evidence and expert consensus/clinical practice guidelines), and contextual criteria, which includes the priority of access to the population, system capacity, appropriate use of the biomarker, and opportunity cost and affordability.

**Table 1:** Multicriteria decision analysis framework adapted to quantify neurofilaments in multiple sclerosis from the MCDA framework.

Quantitative criteria
Multiple sclerosis-related criteria
Severity of multiple sclerosis Population size Unmet needs
Light chain neurofilaments-related criteria
Efficacy/effectiveness: determination of the diagnosis of MS, determination of MS activity, determination of inflammation-associated neurodegeneration and determination of the response to treatment Safety/tolerability Patient-reported outcomes Direct cost Indirect cost Quality of evidence Expert consensus/clinical practice guidelines
Qualitative or contextual criteria
Priority access to the population System capability and appropriate use of neurofilament quantification Opportunity cost and affordability

The information gathered from a literature review was structured into an evidence matrix. The evidence matrix was scored by a multidisciplinary panel of Spanish experts involved in the management of MS. Scores were analyzed quantitatively. Comments and reflections behind experts’ scores were collected in a qualitative manner. To determine the value contribution, the weighting of 98 assessors and decision makers at national and regional level in a previous study from a previous study conducted in Spain was used (24–26, 30–32).

Experts received basic training on reflective MCDA methodology. After the training session in MCDA methodology, the evidence matrix was sent via email to each of the participants for individual scoring. The experts scored the evidence matrix based on current evidence. The results of the participants’ scores were entered into a specific Excel database (27–29), used in the MCDA methodology and adapted to this study. Once the data was analyzed, a second MCDA workshop was held to present the results obtained by the participants and to have a reflective discussion for each of the criteria included in the adapted MCDA framework. Changes in scoring were allowed during the discussion session. This manuscript presents the final scores and primary reflections following the reflective discussion workshop.

### 2.2 Literature review and development of the evidence matrix

The present study was based on a previous literature review, which included articles from 2019 to 2023. The information was complemented with new published evidence by a rapid literature review of biomedical databases, grey literature sources such as the website of the European Medicines Agency and websites of scientific societies and patient associations. The results were synthesized and structured in the evidence matrix following the criteria included in the adapted MCDA framework.

### 2.3 Expert panel design

The study was conducted with a multidisciplinary group of six experts in an online session. They were selected to represent several points of view about the disease and the value of blood-based NfL as a biomarker in MS (two neurologists, one immunologist with extensive knowledge in the management and treatment of MS, one hospital pharmacist, one hospital medical director and one regional healthcare system manager/decision maker).

### 2.4 Data collection and analysis

A non-hierarchical 5-point scale was used (+5 points = high relative importance; 0 point = no relative importance) for the disease and blood-based NfL-related criteria, except for the cost criteria, where 0 represents the best possible value, and 5 the worst possible value. Qualitative or contextual criteria were evaluated according to whether they represented a positive, neutral, or negative impact for the National Health System (33–35). The qualitative criteria score was displayed on a numerical scale of −1, 0, and + 1, representing negative, neutral, and positive impacts, respectively.

The analysis of the results was conducted by obtaining the mean, median, maximum, and minimum, standard deviations, and the number of responses of the experts’ scores for each of the quantitative criteria of the MCDA framework (24–26, 36). For the contextual criteria, the percentages of responses with a positive, negative, and neutral impact were calculated (26, 36, 37).

The value contribution of the determination of blood-based NfL in MS was analyzed with the quantitative criteria of the MCDA framework (disease-related criteria and blood-based NfL-related criteria) (37), and the weights from the validated MCDA reference value framework for drug evaluation and decision making in Spain, which includes weights from 98 evaluators at national and regional level (25), were used. The value contribution (VC_x_) was calculated as the product of the weighting (W_x_) and the standardized scores (S_x_) (25). The overall value contribution is the sum of the individual value contribution of each quantitative criterion:


$$VE=\overset{n}{\underset{x=1}{\sum }}{VC}_{x}=\overset{n}{\underset{x=1}{\sum }}\left(W_{x}\times S_{x}\right)$$

## 3 Results

### 3.1 Performance scores based on evidence and participants’ insights

**Figure 1:**
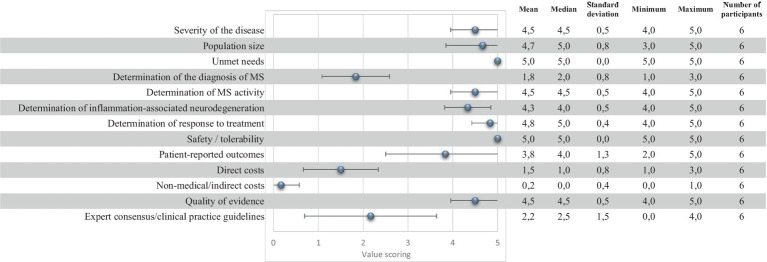
Quantitative criteria scores for the quantification of blood-based neurofilaments light chains in multiple sclerosis.

**Figure 2:**
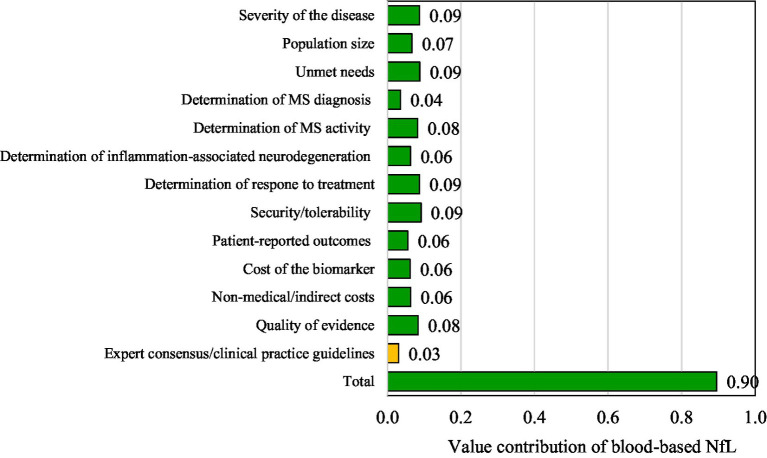
Results of the global value contribution of blood-based neurofilaments light chains as a biomarker in multiple sclerosis.

**Figure 3:**
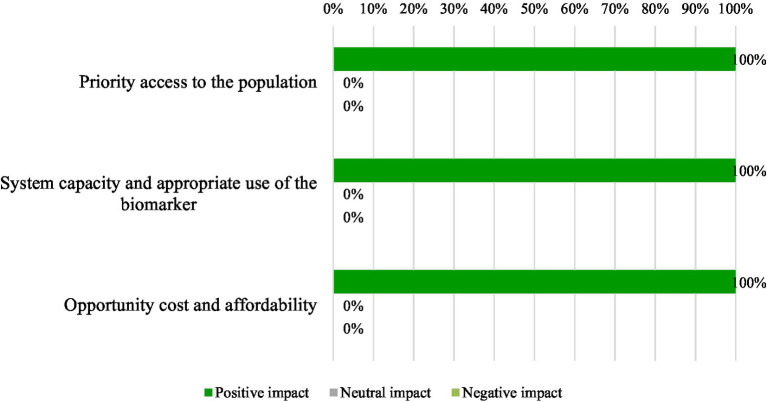
Percentages of experts who would consider the impact of blood-based neurofilaments light chains as a biomarker in multiple sclerosis as positive, negative, or neutral (contextual criteria).

The quantitative criteria of the evidence matrixes were scored, and results were discussed by the panel of experts. The mean, median, standard deviation (SD), minimum (min), maximum (max) and number of responses (n) for each of the analyzed quantitative criteria are shown in Figure 1. The estimated overall value contribution of the quantification of blood-based NfL as a biomarker in MS in Spain is shown in Figure 2. Qualitative or contextual criteria were assessed according to whether they represented a positive, neutral, or negative impact for the National Health System. The outcomes were then converted into percentages, reflecting the proportion of experts supporting each option (Figure 3).

#### 3.1.1 Disease-related criteria

##### 3.1.1.1 Severity of MS

Experts perceived MS as a disease of high severity (mean ± SD: 4.5 ± 0.5) due to the high impact on patients’ quality of life and life expectancy because of the progressive evolution. It was also highlighted that it is one of the most common causes of non-traumatic disability in young and middle-aged adults.

##### 3.1.1.2 Population size

Participants considered that the prevalence of MS is high (mean ± SD: 4.7 ± 0.8) based on the prevalence and increasing incidence of the disease.

##### 3.1.1.3 Unmet needs

Overall, it is considered that there is a very important unmet need in the use of biomarkers in MS (5.0 ± 0.0). Increasing the sensitivity of body fluid biomarkers to anticipate clinical and radiological findings in clinical practice, especially disability progression, is crucial to intervene early and avoid the accumulation of irreversible damage.

#### 3.1.2 Intervention-related criteria

##### 3.1.2.1 Efficacy/effectiveness

It is generally perceived that the determination of blood-based NfL does not stand out for its contribution to the diagnosis of the disease (mean ± SD: 1.8 ± 0.8) as it is not able to differentiate MS from other neurological diseases. However, it will rather be a complementary data to support the diagnosis. Participants agreed that quantification of blood-based NfL is very effective in determining MS activity (mean ± SD: 4.5 ± 0.5), inflammation-associated neurodegeneration (mean ± SD: 4.3 ± 0.5) and in determining response to treatment (mean ± SD: 4.8 ± 0.4).

##### 3.1.2.2 Safety/tolerability

Experts agreed that the safety profile of blood-based NfL extraction is good (mean ± SD: 5.0 ± 0.0). For the quantification of blood-based NfL, a sample of the patient’s blood is required, which is obtained by blood extraction. This test is routinely and recurrently performed, and it is considered low risk and safe. Potential adverse effects are rare, all of them being mild and tolerable.

##### 3.1.2.3 Patient-reported outcomes

Participants agreed that patient-reported outcomes are favorable (mean ± SD: 3.8 ± 1.3). Quantification of blood-based NfL could have a positive impact on the quality of life of patients, thus potentially improving their health outcomes. It was pointed out that the use of this technique in clinical practice may also have a positive psychological impact on patients, as they perceive that they are under more and better control of their disease.

##### 3.1.2.4 Direct cost

The panel agreed that the direct cost of blood-based NfL extraction is relatively low (mean ± SD: 1.5 ± 0.8). The main limitation of the technique is considered the initial investment to purchase the equipment. However, the panel considered that use of blood-based NfL into MS could reduce current costs through the reduction and optimization of the utilization of more expensive tests such as magnetic resonance imaging (MRI) scans and better treatment selection. This can positively influence direct costs minimization. The panel concluded that blood-based NfL have the potential of being cost-effective.

##### 3.1.2.5 Indirect cost

Overall, the panel agreed that the indirect cost associated with blood-based NfL quantification is low (mean ± SD: 0.2 ± 0.4). The experts agreed that this biomarker could decrease the indirect costs currently associated with the management of MS since all the consequences of disability could be reduced in young people thanks to better control of the disease.

##### 3.1.2.6 Quality of evidence

The quality of evidence was perceived as good (mean ± SD: 4.5 ± 0.5) since all studies consistently report the same data, which reinforces the strength of the results. The available publications address all relevant issues (diagnosis, activity, progression and prediction of relapse or evolution). Based on the current evidence, substantial sample sizes and follow-up times allow for robust analysis and statistically significant conclusions.

##### 3.1.2.7 Expert consensus/clinical practice guidelines

The use of blood-based NfL measurement for MS is not adequately reflected in clinical practice guidelines at the time of this study (mean ± SD: 2.2 ± 1.5). Experts agreed, however, that the neurological scientific community clearly endorses the effectiveness of blood-based NfL, despite guidelines not adequately reflecting it.

#### 3.1.3 Contextual criteria

##### 3.1.3.1 Priority access to the population

All experts agreed that the quantification of blood-based NfL would have a positive impact and be aligned with system priorities. Its incorporation into health plans is not yet clearly structured. However, experts believed that it is in line with the implementation of innovative technologies and the transition toward personalized medicine. In fact, there are national plans that aim to promote personalized and precision medicine, such as the Ministry of Health’s 5P Plan (personalized, predictive, preventive, participatory and population-based medicine) (38). The objective of the 5P Plan is to update and expand the infrastructure for health centers in the consolidation of personalized precision medicine, which will allow for a more individualized adaptation of diagnosis and therapeutic or preventive measures.

##### 3.1.3.2 System capability and appropriate use of neurofilament quantification

All participants considered that the National Health System would be ready to introduce blood-based NfL quantification in daily clinical practice for MS in Spain. The only difficulty is the purchase, installation, and establishment of the routine in the laboratory for its performance. It is not a technique that causes a high healthcare impact, since once it is established. Thus, it is just another determination without major technical complications.

##### 3.1.3.3 Opportunity cost and affordability

Experts agreed that the quantification of blood-based NfL would have a positive impact on the opportunity cost to the National Health System because it is a minimally invasive test, and beyond the initial purchase of the equipment, the associated direct costs are low. Quantification of blood-based NfL could incur a reduction in the costs associated with MS (both direct and indirect), improving its affordability.

### 3.2 Global value contribution of blood-based NfL in MS

The criteria scores were weighted to estimate the overall value contribution of the quantification of blood-based NfL as a biomarker in MS in Spain (Figure 2). The result was +0.90 (scale between 0 and + 1; being +1 maximum value contribution). The greatest contribution to the overall value came from disease severity, unmet needs, determination of response to treatment and security/tolerability, all of them with a score of +0.09.

## 4 Discussion

The value contribution of blood-based NfL as a biomarker in MS was assessed through reflective MCDA by a multidisciplinary panel of stakeholders involved in the management of MS and decision-making in Spain.

MS is perceived as a severe disease with high morbidity and high impact on life expectancy due to disease activity and neurodegeneration. Patients experience a significant impairment in their quality of life because of the disability associated with the disease (3, 39). MS is also considered to have high prevalence, affecting over 2.8 million worldwide (1, 2, 40, 41). Unmet needs have been identified in the determination of disease activity, measurement of neurodegeneration, and response to treatment of patients with MS at an early stage. Traditionally, clinical and radiological variables have been used to assess these outcomes, but they usually reflect an already established neurological damage. Thus, a biomarker capable of determining both inflammatory activity and neurodegeneration and identifying patients at risk of disease progression at an early stage is urgently required (9, 10). It is also essential to promptly initiate optimal treatment, continuous monitoring of therapeutic response, anticipation of treatment decisions, and achieve better therapeutic individualization. The final aim is to improve the quality of life for both patients and caregivers (5, 9, 13–16, 42).

The results of this study suggest that quantification of blood-based NfL could be used to determine disease activity, complementing clinical and radiological information. In addition, measurement of blood-based NfL may be used to establish inflammation-associated neurodegeneration, identifying patients who are entering the neurodegenerative stages of the disease. It has also been considered that blood-based NfL, as a biomarker of neuronal damage, could provide value in determining treatment response at an earlier stage than clinical or radiological variables. The reason is that blood-based NfL reflect neuronal inflammation at a deeper level than MRI or clinical manifestations. Its integration into clinical practice could enhance the process of selecting appropriate treatments, evaluating treatment responses, and ensuring ongoing monitoring of therapeutic efficacy. This would involve identifying patients who require treatment due to worsening disease progression, patients who can safely discontinue treatment due to disease improvement, or patients at risk of treatment failure or severe adverse reactions, thereby indicating the need for a change in treatment (9, 16, 43). Blood-based NfL is not specific to MS, which implies that it is not a diagnostic marker for MS (10). Nevertheless, measuring blood-based NfL levels could still have value in providing complementary data to enhance the sensitivity and specificity of MS diagnosis. This is especially relevant in patients with clinically isolated syndrome or in the early stages of the disease that do not yet meet established diagnostic criteria (5, 9, 13, 14, 16, 17, 42). Participants consider blood-based NfL as a highly secure biomarker. To quantify blood-based NfL, a patient’s blood sample is required, obtained through blood extraction (5). This test is routinely and regularly performed, considered low-risk and safe, with potential adverse effects being rare and generally mild and tolerable, which contributes to a good safety profile of the intervention. In fact, the risks associated with blood extraction are perceived to be negligible. Given that patients with MS undergo blood tests every 3–6 months (6), experts did not consider that blood-based NfL determination could introduce any additional risk or extra costs.

According to the experts, the quantification of blood-based NfL may have the potential to positively influence the overall quality of life of patients with MS by enabling more effective disease monitoring, thereby enhancing the possibility of improved health outcomes. Moreover, it could help patients maintain their emotional well-being, and integrating blood-based NfL quantification into MS management may provide significant benefits, as they may perceive a better control of their disease. It can give patients a greater sense of security as they could know they do not have neuronal inflammation, despite the uncertainties associated with their conditions.

Regarding the costs associated with the use of blood-based NfL as a biomarker, experts considered that it could result in both direct and indirect cost savings. The panel considered that incorporating blood-based NfL into MS clinical practice could have the potential to reduce the need for more expensive tests, such as MRI scans, thereby optimizing resource utilization. Considering that patients with MS already undergo periodic blood tests, the integration of blood-based NfL would not impose any additional burden on patients or hospitals in this regard. Furthermore, the expert panel emphasized the importance of refining treatment selection and the potential value of blood-based NfL in facilitating the early identification of non-responders, ultimately mitigating medical costs linked to ineffective treatments (44). The panel also agreed on the potential value of blood-based NfL in reducing indirect costs associated with MS. Quantification of blood-based NfL provides more information about blood-based NfL, facilitating a better control and likely reducing indirect costs associated with disease management. It has the potential to act as a preventive measure against disease progression and mitigate the overall consequences of disability, likely enhancing productivity among patients. This is especially relevant in young patients (45). The main economic limitation identified in blood-based NfL quantification was the initial investment required for laboratory equipment. However, it was stated that as the technique becomes more widely adopted, the costs associated with equipment purchase could decrease. Indeed, some hospitals already possess the technology required, with a potentially cost-effective analysis per sample. Overall, the panel concluded that with the upcoming development and investment, the long-term benefits and potential cost savings make blood-based NfL extraction a viable and economical choice as a biomarker in MS.

The incorporation of blood-based NfL measurement for MS is inadequately represented in clinical practice guidelines in Spain or globally at the time of this study (39, 46). The quantification of blood-based NfL is a novel and innovative approach that is currently being explored for potential inclusion in broader clinical recommendations. Nevertheless, experts unanimously affirmed that, within the neurological scientific community, there is clear endorsement of the utility of blood-based NfL quantification in MS despite this limited inclusion in guidelines.

However, it is important to note that, when evaluating individual blood-based NfL levels, it is necessary to consider several factors, including the influence of various pathophysiological variables such as renal function, blood volume, and body mass index (BMI). Understanding how these factors can impact blood-based NfL levels is essential for accurate interpretation of results and make more informed decisions (18). Thus, the use of a standardized score (z-score), indicating the age and BMI-adjusted standard deviations of blood-based NfL levels from a dataset of healthy donors (16), improves interpretation of blood-based NfL concentrations at an individual level. Potential modifications of this z-score might be warranted in the future, depending on whether additional variables influencing blood-based NfL values are further described.

MCDA methodology has been employed in recent studies to assess the value contribution of a health technology in a range of medical conditions. Additionally, it functions as a valuable tool for Health Technology Agencies and pharmacotherapeutic committees, facilitating evaluations and decision-making processes. Using MCDA methodology enables a comprehension of the perceived value of a new health technology, achieved through the scoring of the evidence matrix, considering a diverse range of value attributes (33–35). It is therefore understandable that MCDA methodology is becoming increasingly popular to support healthcare decision-making, particularly in complex cases (26–28, 31, 32).

The present study is not exempt from some limitations (35). First, the participation of a relatively small number of experts may introduce potential bias. The decision to opt for a modest panel size in the MCDA exercises was deliberate, aiming to foster active participation in group discussions and encourage the sharing of diverse perspectives, facilitating a more in-depth analysis of the various value criteria under consideration. Second, the results may be affected by the composition of the expert panel, their value judgements, and their experience. Participants received training on MCDA methodology prior to the individual scoring work and discussion session to mitigate the risk of expertise bias.

To our knowledge, this is the first study to apply MCDA methodology to establish the overall value contribution of blood-based NfL as a biomarker in MS. The findings suggest that blood-based NfL quantification in MS represents a high-value option for determining disease activity, neurodegeneration, and response to treatment, from the experts’ perspective. MCDA has demonstrated to be useful to compare the value of health technologies in MS, allowing analysis and reflective discussion in a systematic, objective, pragmatic and transparent way from the point of view of key stakeholders involved in the management of MS.

## Data availability statement

The raw data supporting the conclusions of this article will be made available by the authors, without undue reservation.

## Author contributions

EM: Writing – review & editing. PR: Writing – review & editing. IS: Writing – review & editing. AR-A: Writing – review & editing. MM-M: Writing – review & editing. AÁ: Writing – review & editing, Conceptualization. EG-A: Conceptualization, Writing – review & editing. JM: Conceptualization, Writing – review & editing. JS: Writing – review & editing. ÁC: Writing – original draft. LV: Writing – review & editing.
